# Identification of donor *Bacteroides vulgatus* genes encoding proteins that correlate with early colonization following fecal transplant of patients with recurrent *Clostridium difficile*

**DOI:** 10.1038/s41598-023-41128-y

**Published:** 2023-08-29

**Authors:** Hyunmin Koo, Casey D. Morrow

**Affiliations:** 1 Department of Genetics, Hugh Kaul Precision Medicine Institute, University of Alabama at Birmingham, Birmingham, AL USA; 2 Department of Cell, Developmental and Integrative Biology, Hugh Kaul Precision Medicine Institute, University of Alabama at Birmingham, Birmingham, AL USA

**Keywords:** Metagenomics, Microbiome, Dysbiosis

## Abstract

Due to suppressive antibiotics, patients with recurrent *Clostridium difficile* have gut microbial communities that are devoid of most commensal microbes. Studies have shown that most of the failures using fecal microbe transplantation (FMT) for recurrent *C. difficile* occur during the first 4 weeks following transplantation. To identify features of donor *Bacteroides vulgatus* that lead to early colonization, we used two data sets that collected fecal samples from recipients at early times points post FMT. The first analysis used the shotgun metagenomic DNA sequencing data set from Aggarwala et al. consisting of 7 FMT donors and 13 patients with recurrent *C. difficile* with fecal samples taken as early as 24 h post FMT. We identified 2 FMT donors in which colonization of recipients by donor *B. vulgatus* was detected as early as 24 h post FMT. We examined a second data set from Hourigan et al. that collected fecal samples from *C. difficile* infected children and identified 1 of 3 FMT that also had early colonization of the donor *B. vulgatus*. We found 19 genes out of 4911 encoding proteins were unique to the 3 donors that had early colonization. A gene encoding a putative chitobiase was identified that was in a gene complex that had been previously identified to enhance colonization in mice. A gene encoding a unique fimbrillin (i.e., pili) family protein and 17 genes encoding hypothetical proteins were also specific for early colonizing donors. Most of the genes encoding hypothetical proteins had neighboring genes that encoded proteins involved in mobilization or transposition. Finally, analysis of 42 paired fecal samples from the human microbiome project (HMP) found no individuals had all 19 genes while 2 individuals had none of the 19 genes. Based on the results from our study, consideration should be given to the screening of FMT donors for these *B. vulgatus* genes found to enhance early colonization that would be of benefit to promote colonization following FMT.

## Introduction

Fecal microbial transplant (FMT) has been shown to be highly effective for the treatment of patients with recurrent *Clostridium difficile*^1–3^. The FDA has recently approved FMT for the treatment of recurrent *C. difficile*^4,5^. For patients with recurrent *C. difficile*, FMT was used primarily as a last resort following the failure of standard therapy that consists of multiple rounds of suppressive antibiotics that can nearly eliminate the recipient commensal microbial community^6–9^. The degree of recipient strain microbial community depletion, because of the several rounds of suppressive antibiotics, was also an important consideration for donor colonization^10^.

To better characterize the extent of donor microbial colonization in recipients following FMT, several studies have used metagenomic sequencing coupled with new informatic tools that allowed the resolution of the microbial community at the strain level^6,11–13^. We, and others, have shown that recurrent *C. difficile* patients who had FMT can result in the stable long-term colonization of donor microbial strains for up to 2 years post FMT, the longest time examined^6,7^. The dynamics of early colonization following FMT though, are less defined since most studies do not collect samples (or report) on the microbial composition. Interestingly, previous studies have reported that FMT failure occurs most of the time during the first 4 weeks following FMT suggesting this is a critical time needed to establish the stable microbial community structure^14,15^.

In this study, we have investigated the dynamics of microbial community colonization at early times post FMT. To do this, we have used publicly available data sets that reported the microbial composition in the recipient at multiple early times post FMT^16,17^. Using our Window-based single-nucleotide variant (SNV) similarity (WSS) strain tracking analysis^6,18–22^, we have focused the analysis on *B. vulgatus*, which is one of the most prevalent commensal fecal microbes in humans^23,24^. Furthermore, the *Bacteroides such as B. vulgatus,* are considered to be models in which to study bacterial colonization in the host gastrointestinal tract^25^. Indeed, a previous study in mice identified a commensal colonization factor (CCF) gene complex that Bacteroides use for physical interactions with the host that mediate stable and resilient gut colonization^26^. Our study demonstrates the presence of donor *B. vulgatus* in the feces of three donors as early as 24 h post FMT from Aggarwala et al. and 2–7 weeks post FMT from Hourigan et al. Analysis of the common genes between the three donors revealed that only 19 were in common out of 4911 genes encoding known and hypothetical proteins. The result from our analysis supports the screening of donor *B. vulgatus* for this gene consortium to enhance colonization following FMT.

## Results

Aggarwala et al. and Hourigan et al. have recently described the analysis of FMT given to patients with recurrent *C. difficile*^16,17^. In both studies, fecal samples were taken at early times (24 h for Aggarwala et al. and 2–7 weeks for Hourigan et al.) and subjected to metagenomic DNA sequencing. The recipients for each study had undergone standard antibiotic therapy consisting of multiple rounds of antibiotic therapy. In both Aggarwala et al. and Hourigan et al., the microbial community of the recipient pre-transplant was reduced or devoid of the commensal microbes found in a healthy fecal microbial community^16,17^ (information on sequence reads downloaded for this study is listed in Supplemental Table 1).

**Figure 1:**
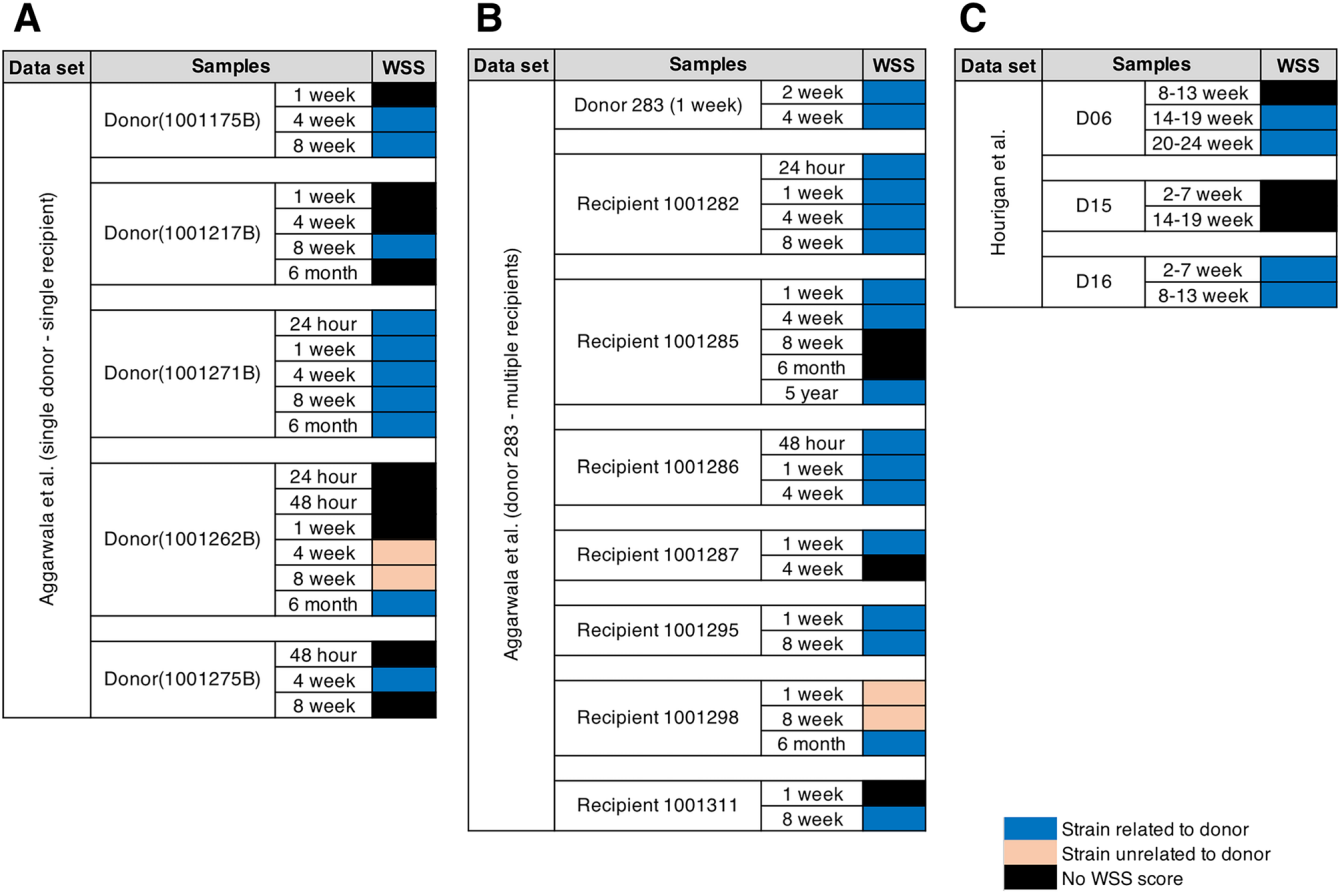
WSS analysis identified early and late colonization phenotype. Summarized WSS scores for donor-recipient pairs from (**A** and **B**) Aggarwala et al. and (**C**) Hourigan et al. The WSS scores were observed comparing the donor’s sample to paired recipient’s post-FMT samples. All samples used for the analysis were listed in Supplemental Table 1. The WSS scores per each donor-recipient pair were grouped into different color boxes (see the figure key). Only *B. vulgatus* species was included to represent WSS scores for each donor-recipient pair. The black boxes indicate the *B. vulgatus* that we were unable to determine relatedness due to the any sample in pairs not satisfying the criteria of WSS analysis (minimum coverage > 30% and average depth > 3.5). In addition, the samples with the orange boxes were not related to either donor or recipient (i.e. below the cut-off value)^6^.

The metagenomic sequence reads obtained from Aggarwala et al. and Hourigan et al. were analyzed using the WSS analysis^6^. Using the WSS, we first analyzed the Aggarwala et al. recipients post FMT to determine the presence of the donor-related microbes. First, 5 donor-recipient pairs were analyzed that collected samples as early as 24 h post FMT (Fig. 1A). We focused our analysis on *B. vulgatus* which we knew from the Aggarwala et al. results were present in the recipient post-transplant^16,17^. We found two distinct patterns with respect to the WSS result. In the first pattern, the FMT with Donor 1001271B had donor *B. vulgatus* microbes in the recipient post-transplant fecal sample at 24 h that remained stable at different analysis times for 6 months. In the second pattern, donors of 1001175B, 1001217B, 1001262B and 1001275B, did not have a WSS score for *B. vulgatus* until at least 4 weeks post-transplant. We also note that Donors 1001217B and 1001275B had no WSS scores at later times (8 weeks and 6 months). In a second set of experiments described by Aggarwala et al., a single donor, 283B, was used for FMT in multiple recipients (Fig. 1B). In 5 of the 7 FMT, we found the recipients post-transplant had donor related *B. vulgatus* at times before 4 weeks while the donor 283B *B. vulgatus* strain was detected on the feces at later times for recipients 1001298B and 1001311B suggesting some variability in the FMT that might be related to the condition of the recipient’s gastrointestinal tract. In the second data set, Hourigan et al., fecal samples were taken at early times (2–7 weeks) and later times (8–13, 14–19 or 20–24 weeks). The recipient post FMT using donor D15 had *B. vulgatus* as early as 2–7 weeks, while those using D06 had no WSS score at this early time while D15 had no WSS score at any times examined after FMT (Fig. 1C).

**Figure 2:**
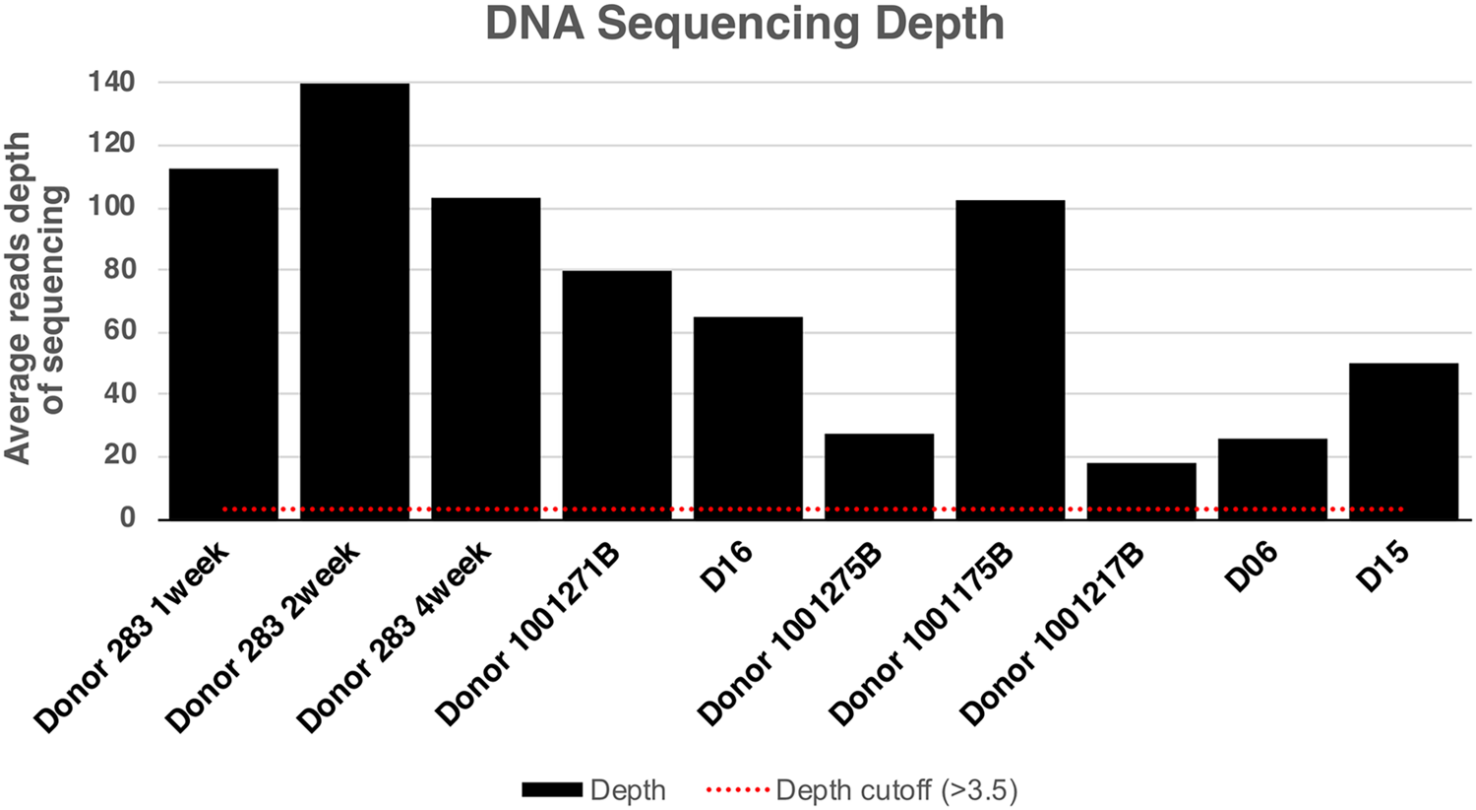
DNA Sequencing depth of donors. For each donor from Aggarwala et al., and Hourigan et al. data set, sequence depth against reference *B. vulgatus* genome implemented in our WSS database was calculated and shown in here. Sequence depth cutoff value for WSS analysis is > 3.5. A detailed sequence depth and coverage for each sample was shown in Supplemental Table 2.

In our previous study, we found that the WSS score was dependent upon both the sequencing depth and coverage against to a reference genome, but not necessarily the relative abundance of *B. vulgatus* in individual fecal samples^6^. A sequencing depth of 3.5 and a minimum coverage of 30% are needed to satisfy the requirements for obtaining the WSS score^6^. The “No WSS score” for the recipient’s post-transplant from Aggarwala et al. and Hourigan et al. at the early times were all due to either a depth lower than 3.5 or/and coverage lower than 30% (Supplemental Table 2). Detailed information regarding sequence depth and coverage of the *B. vulgatus* was shown in Fig. 2 and Supplemental Table 2. We found that the early donor phenotype (283B, 1001271B and D16) had a similar sequencing depth seen for donor 1001175B (a late colonizer). It was clear though that all the donor fecal samples had a sufficient read depth/coverage (denoted by the red line). Thus, the differences in the donor read depth did not explain the differences in *B. vulgatus* colonization of recipients. An additional possibility would be that the differences in the donor *B. vulgatus* colonization could be explained by different replication rates^27^. However, using a Growth Rate Index (GRiD) analysis^19,27^, we found no significant differences between the different fecal donors (Supplemental Table 3). Finally, in our previous studies, we used the WSS analysis to show that in 42 paired fecal samples from the human microbiome project (HMP) that *B. vulgatus* from different individuals were not related while the *B. vulgatus* from the same individual taken at different times was related^6^. Similarly, a WSS analysis of the donors from Aggarwala et al. and Hourigan et al. also found that donors were not related while donor 283B samples taken at different times were related (Supplemental Table 4).

Based on our WSS analysis of recipients post FMT, we operationally identified for our study the *B. vulgatus* of donor 1001271B and donor 283B (from Aggarwala et al.) and donor D16 (from Hourigan et al.) as an early colonization phenotype. We found that *B. vulgatus* several donors had a colonization phenotype that manifested as a delay in the appearance of the donor *B. vulgatus* in the recipient post FMT (1001175B, 1001262B and D06). In addition, several of these donors (Donor 1001217B and 1001275B) had a pattern of delayed appearance of donor *B. vulgatus* in the recipients as determined by WSS at later times. Finally, we identified one donor (D15) that was not detected in the recipient post FMT, even though we confirmed this donor had *B. vulgatus* (Fig. 2, Supplemental Table 2).

**Figure 3:**
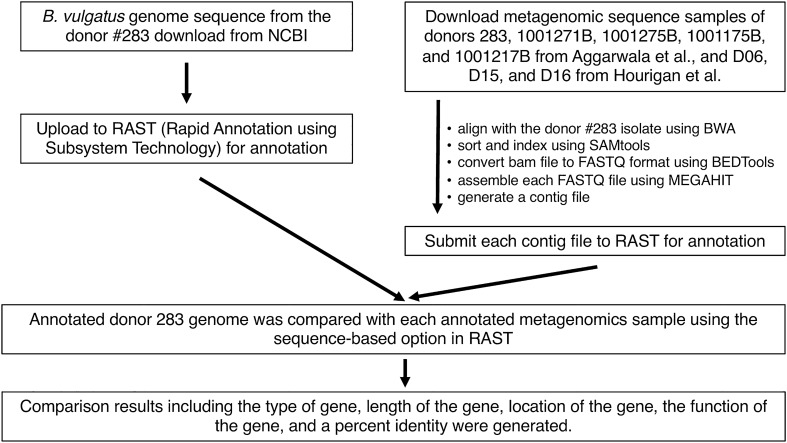
Summary of comparative gene analysis between early versus late colonizers. For comparative gene analysis between early vs. late colonizers, we have designed our approach to align against assigned reference genome, assemble reads, and annotate each sample. All metagenomic data sets were downloaded and aligned with *B. vulgatus* 1001283B150304-161114-D8. Then aligned reads were used for assembly process and then annotated using RAST server. A complete list of genes that observed from this analysis was shown in Supplemental Table 6.

To investigate the reason for these differences in the colonization of the donor *B. vulgatus* in the recipient post FMT, we designed an informatics approach to compare the gene content (Fig. 3). Our strategy took advantage of the availability of the sequenced genome clone for the donor 283B *B. vulgatus* that was provided by Aggarwala et al.^16^. Using this as a reference genome, we uploaded it to Rapid Annotation using subsystem technology (RAST). We also downloaded the metagenomic sequences from samples of donors 283B, 1001271B, 1001275B, 1001175B, and 1001217B from Aggarwala et al., and D06, D15, and D16 from Hourigan et al. Using the procedure outlined in Fig. 3, we analyzed the gene function, length and determined the location in the *B. vulgatus* genome. We found 19 genes out of 4911 identified genes that were significantly different between early and late colonizers (Table 1 and Supplemental Table 5 which contains the statistical analysis for all 4911 genes).

**Table 1:** Statistical analysis between early and late donors for a total of 4911 genes.

Gene ID	Length (aa)	Location in reference genome	Function	*p*-values	*p*-values (FDR correction)
peg.3253	46	64.238–64.101	Hypothetical protein	6.24E−08	1.61E−05
peg.4545	65	5.283–5.477	Hypothetical protein	6.24E−08	1.70E−05
peg.4546	70	5.943–5.734	Hypothetical protein	6.24E−08	1.80E−05
peg.4547	42	5.995–6.120	Hypothetical protein	6.24E−08	1.92E−05
peg.4548	47	6.189–6.329	Hypothetical protein	6.24E−08	2.04E−05
peg.4549	136	6.825–6.418	Hypothetical protein	6.24E−08	2.19E−05
peg.4632	231	9.286–9.978	Hypothetical protein	6.24E−08	2.36E−05
peg.4633	394	9.993–11.174	Hypothetical protein	6.24E−08	2.55E−05
peg.4634	258	11.171–11.944	Hypothetical protein	6.24E−08	2.79E−05
peg.4635	187	12.033–12.593	Hypothetical protein	6.24E−08	3.06E−05
peg.4625	206	2.840–3.457	Hypothetical protein	6.24E−08	3.40E−05
peg.4624	114	2.502–2.843	Hypothetical protein	6.24E−08	3.83E−05
peg.925	451	169.520–170.872	putative chitobiase	6.24E−08	4.38E−05
peg.3499	569	47.276–45.570	Fimbrillin family protein	6.24E−08	5.11E−05
peg.2053	224	92.891–93.562	Hypothetical protein	6.24E−08	6.13E−05
peg.918	41	161.693–161.571	Hypothetical protein	6.24E−08	7.66E−05
peg.3015	260	12.096–11.317	Hypothetical protein	6.24E−08	0.000102142
peg.3014	155	11.283–10.819	Hypothetical protein	6.24E−08	0.000153214
peg.3504	113	51.067–50.729	Hypothetical protein	6.24E−08	0.000306427

The gene differences between early (283B, 1001271B, and D16) and late (1001275B, 1001175B, 1001217B, D06, and D15) donors were compared through ANOVA with Benjamini–Hochberg FDR correction using STAMP. Only significant *p*-values (< 0.05) and corrected *p*-values (< 0.05) are included in this table (see Supplemental Table 5 for the entire statistical analysis results).

**Figure 4:**
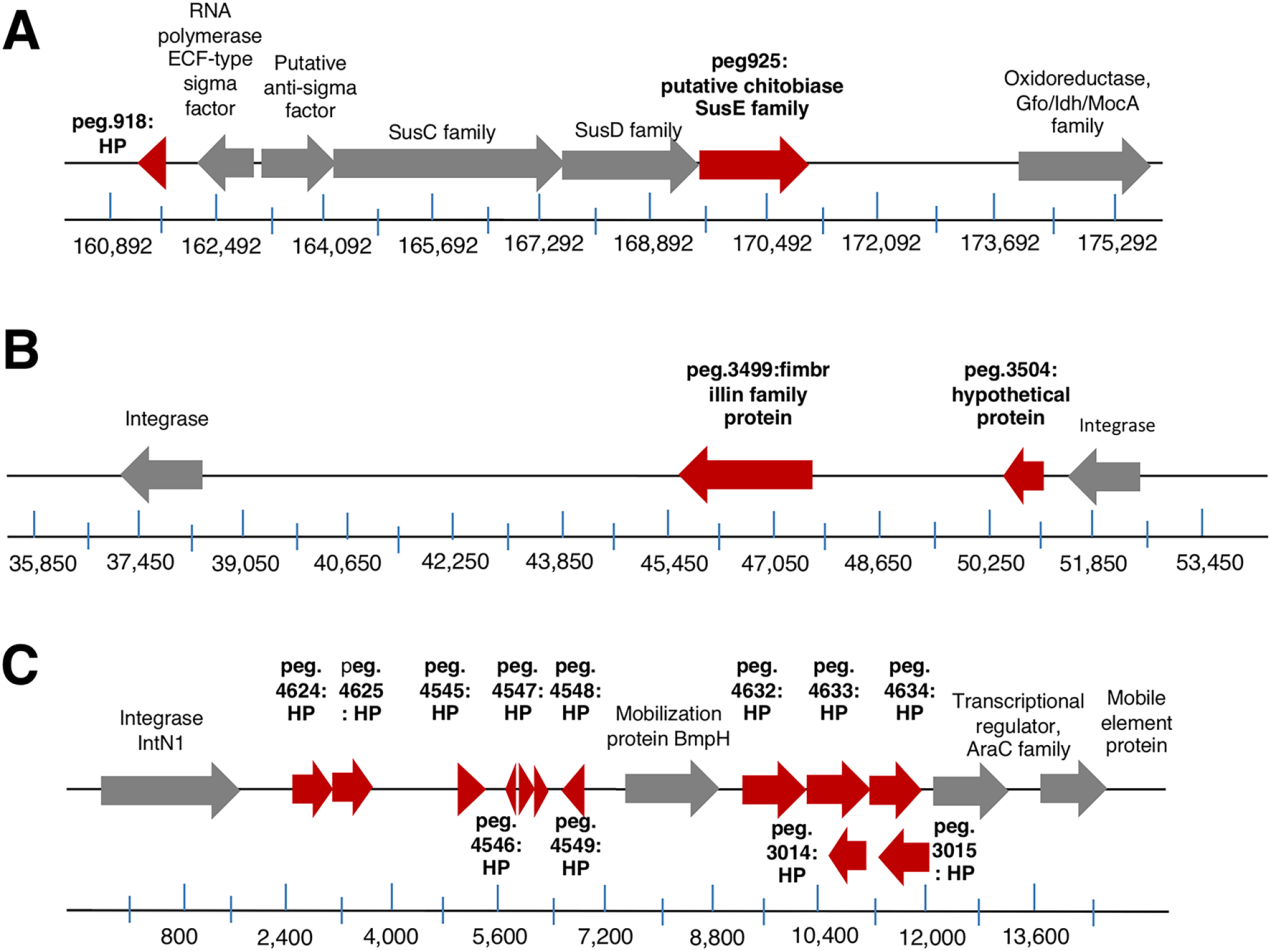
Genome location of the genes found commonly in early colonizers. To visualize genome location for 19 genes that was commonly found in early colonizers, we have used Rapid Annotation using Subsystem Technology (RAST) server (https://rast.nmpdr.org/)^46^. Detailed analysis can be found in “Materials and methods” section. Genes were closely located to (**A**) putative chitobiase, (**B**) Fimbrillin protein, and (**C**) hypothetical proteins were shown in red and they were present only to the early donors. The genes in gray were found in both the early and late donors (Supplemental Table 6).

Within the 19 genes, 2 of the genes were identified as a putative chitobiase and fimbrillin (i.e. pilli) family while 17 of the 19 genes were listed as hypothetical proteins of lengths ranging from 41 to 394 amino acids. The putative chitobiase was located in a cluster of genes including putative SusC and SusD proteins with genes encoding sigma and anti-sigma factors (Fig. 4); note that both early and late donors had genes for the putative SusC and SusD proteins with genes encoding sigma and anti-sigma factors (Supplemental Table 6). This configuration of genes has been previously described in *B. fragilis* and *B. vulgatus* and shown to enhance colonization when transplanted into mice with a reduced commensal microbial community^26^. The fimbrillin gene is localized in the same vicinity as one of the genes encoding peg 3504 hypothetical protein (Fig. 4). Genes encoding integrases were located 3′ and 5′ from the fimbrillin and peg 3504 in the donor 283B *B. vulgatus* genome. We also found that many of the genes encoding hypothetical proteins formed a gene cluster (Fig. 4). Similar to the fimbrillin gene, we also found genes encoding some of these hypothetical proteins that were implicated in horizontal gene transfer or transposition^28–31^.

**Figure 5:**
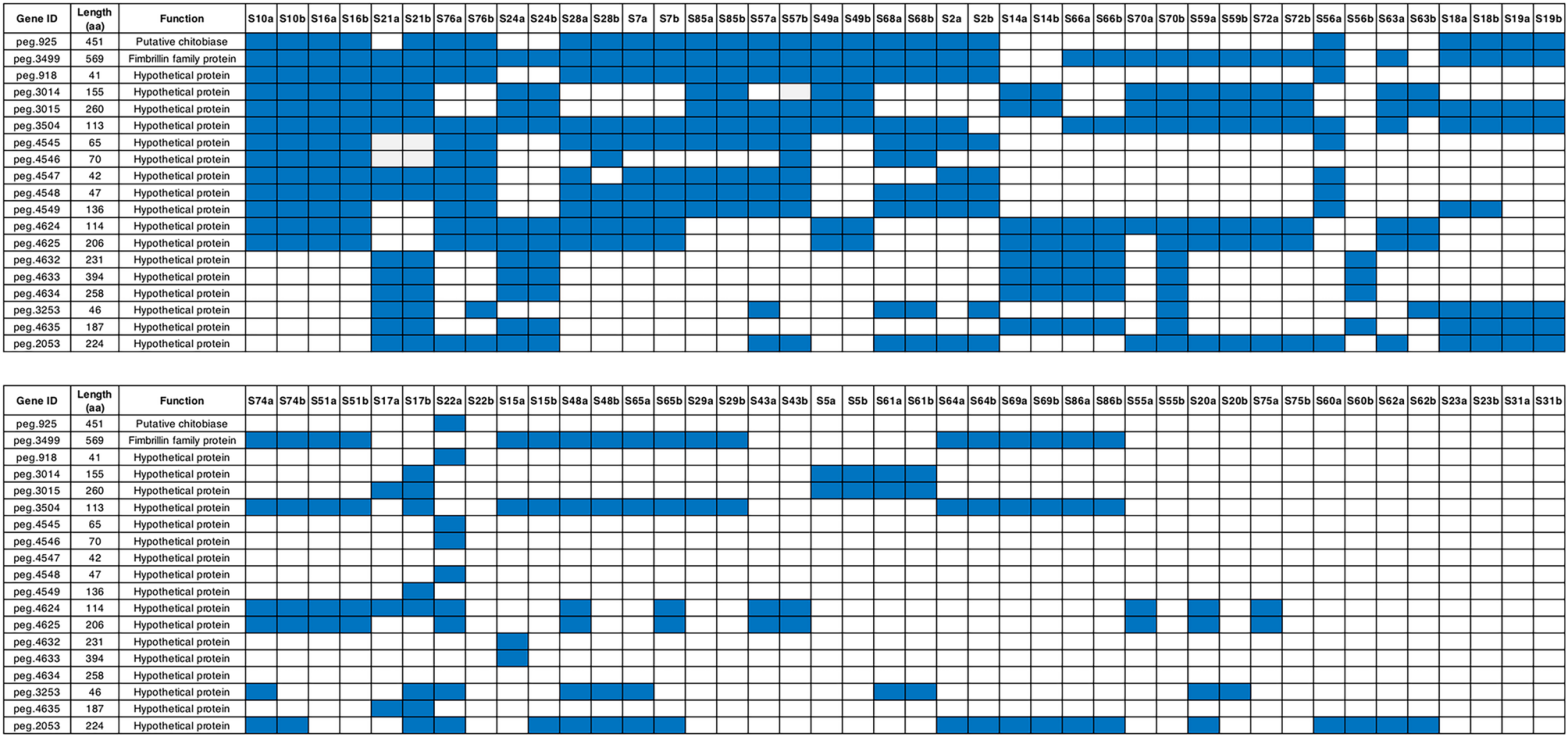
A list of genes for HMP data set. We have analyzed the 42 paired sample data set from the HMP. Those samples which contained the 19 genes are noted (present in a blue box, absent in a white box). S23 and S31 did not have any of the 19 genes. There were 17 sample pairs where the pattern of the 19 genes varied between the two time points (a and b) for the same individual.

We next determined the distribution of the 19 genes in a larger set of samples, the HMP data set consisting of 42 paired samples taken at different times up to 6 months apart^32^ (Fig. 5). We found no samples in the HMP data set had all 19 genes and sample pairs were identified that have the putative chitobiase and fimbrillin genes, with the varied presence of the hypothetical proteins. We also identified 2 sample pairs that did not contain any of the 19 genes consistent with the late colonizers 1001275B, 1001175B, 1001217B, D06, and D15. We found 17 sample pairs where the pattern of the 19 genes varied between the two time points for the same individual. Only 3 pairs of the 17 had differences in the two samples for encoding genes for chitobiase and fimbrillin. Collectively, the results of our analysis then show the presence of all 19 genes in the samples from the HMP database, although none of the pairs in this data set contained all the 19 genes that were found in donors 283B, 1001271B and D16.

## Discussion

In this study, we utilized two data sets that were unique because they analyzed the fecal composition of recipients at early times post FMT. We identified the three donors from both studies in which their *B. vulgatus* was detected in recipients early after FMT. The gene content of the *B. vulgatus* of these early donor phenotypes differed from the late donor phenotypes by the presence of 19 genes encoding proteins. Our results provide a rationale for the analysis of donor *B. vulgatus* for these 19 genes to enhance the early colonization of *B. vulgatus* following FMT.

FMT has been used with great success to restore the microbial community of patients with recurrent *C. difficile*^1–3^. To further delineate the dynamics of microbial community reconstruction following FMT, we used two published data sets that followed the colonization of the recipient early post FMT^16,17^. We focused on the analysis of the donor *B. vulgatus* colonization during the first 4 weeks after FMT because it is one of the more abundant microbe species found in most human feces^25,33^. Furthermore, as suggested by Wexler and Goodman, the interaction between Bacteroides microbes could also extend to others in the gut ecosystem to help establish the normal gut microbial community^25^. From the two separate studies, we identified three donors with a phenotype following FMT that *B. vulgatus* was detected early as 24 h in the recipient following FMT. This result could not be simply explained by limiting amounts of *B. vulgatus,* in the donors as shown from the comparison of the sequencing depth of late phenotype Donor 1001175B was greater than that of two early donor phenotypes (1001271B and D16). In addition, we found no significant differences in the replication potential of the donor *B. vulgatus* used in the FMT that was examined.

To further delineate differences between the early and late phenotype donors, we compared genes encoding proteins between the different donors. For these studies, we made use of the whole genome sequencing reads from donor 283B *B. vulgatus* isolate as a reference genome in combination with bioinformatic tools to compare the genes of donor 283B with early colonizing donors 1001271B and D16 along with the late colonizing donors 1001175B, 1001275B, 1001217B, D15 and D06. We identified 4911 genes encoding known and hypothetical proteins, which is consistent with what is known for other strains of *B. vulgatus*^34^. We found that 19 genes encoding proteins were common between donors 283B, 1001271B and D16 that were not found in the late colonizing donors. One of these genes, a putative chitobiase, was found next to genes encoding SusD, SusC, putative anti-sigma factor, and RNA polymerase ECF-type sigma factor genes. A previous study identified these genes as components of a commensal colonization factor (*ccf*) complex that has been found in *B. fragilis* and *B. vulgatus*^26^. The *ccf* system was found to promote specific interaction with the host that facilitates stable and resilient colonization in mice^26^. Our results then, provide support that the *ccf* system might also function in humans to enhance the colonization of *B. vulgatus*. A second unique gene encoding a fimbrillin family protein was also identified. Fimbrillins (or pili) are protein polymers that protrude from the surface of microbes and serve as anchors for microbial interaction with the host cells and have been identified in Bacteroides^35^. Based on their known functions, the identification of both the chitobiase (and subsequently a complete ccf) and fimbrillin proteins supports the involvement of these proteins in *B. vulgatus* colonization.

Most of the genes identified that were unique to donors 283B, 1001271B and D16 were classified as hypothetical proteins that to date had no identified function. We mapped the larger hypothetical proteins to the 283B genomes and found that, in general, there were localized genes involved in mobilization (e.g. mobile elements) and transposition^28–31^. To further explore this result, we analyzed the 42-paired samples from the HMP data set for investigating the 19 genes. We found that none of the samples had the complete set of the 19 genes that were found in 283B, 1001271B and D16. We acknowledge a weakness of our study that since none of the HMP pairs had the full 19 genes, we do not know whether any of the HMP pairs would have an early colonizing phenotype in FMT. Based on our analysis, we would assume that the *ccf* and fimbrillin genes would suffice but to resolve this issue, additional FMT for recurrent *C. difficile* in humans using early analysis times post FMT would need to be done.

Finally, the question to be asked then is what might be the benefits of screening the donor *B. vulgatus* for the early colonization genes? Based on our analysis of Aggarwala et al. and Hourigan et al., most probably *B. vulgatus* of donors with none of the 19 genes would be late colonizers. Additional studies would be required to confirm that these donors would have a greater propensity for FMT failure. A further application for the screening would be for use within the hospital setting or following chemotherapy or transplants, which are known to involve medication that can disrupt the normal microbial gut flora^22,36^. The capacity to rapidly restore the *B. vulgatus* community in these patients would be important to reduce the risk of infection by pathogens or antibiotic-resistant microbe pathogens that could impact overall health^37,38^.

## Materials and methods

### Publicly available data sets used in this study

In this study, we used 3 publicly available metagenomic data sets from (1) 7 FMT healthy donors and 13 recurrent *Clostridium difficile* infection (CDI) FMT recipients^16^, (2) 9 FMT healthy donors and 9 recurrent *Clostridium difficile* infection FMT children^17^, and (3) the NIH Human Microbiome Project (HMP)^32^. For the Aggarwala et al. data set, we used 6 FMT healthy donors with 12 recurrent CDI FMT recipients due to repeat FMT conducted on donor 1001099B with its paired recipient. From Hourigan et al. data set, we selected 3 healthy donors with 3 recurrent CDI FMT children because donors D07 and D17 had related *B. vulgatus* strain when each donor sample was compared with the pre FMT recipient sample. For the HMP data set, 42 individual samples that were previously used to establish our WSS analysis were selected to run the analysis^6,32^.

### Sequence reads and processing

Sequence reads from Aggarwala et al., Hourigan et al., and the HMP data set were downloaded and used for pre-processing steps including filtering low quality reads using Trimmomatic^39^. After the pre-processing step, the resultant sequence reads from three data sets was used for the downstream analysis.

### WSS analysis

For Aggarwala et al. and Hourigan et al. data sets, pairwise comparisons were conducted on each donor’s sample compared to its recipient pre and post FMT samples using WSS analysis^6,18–22^. For the HMP data set, pairwise comparisons were also performed on each individual’s sample compared to the same individual’s sample collected at different time points. We have additionally conducted a WSS analysis to compare all possible pairs of donors used in this study. For the WSS analysis, the processed reads were aligned to the 93 microbial reference genomes which were previously established based on the HMP dataset^6,23^ using the Burrows-Wheeler aligner (BWA) tool BWA-MEM^40^. Multi-sample SNVs for each given reference genome were measured among all samples for each sample using the Genome Analysis Toolkit (GATK)^41^. The resultant multi-sample Variant Call Format (VCF) files were then used for pairwise comparisons between every possible pair of samples to calculate their overall genome-wide SNV similarity for each microbial species. Any samples having low sequence coverage (< 30%) and low sequence depth (< 3.5) against their given reference genome were excluded from the pairwise comparisons^6,18–22^. After quality-based filtering processes, *B. vulgatus* species that was able to provide the WSS score was selected from each data set. To determine a related strain, a WSS score for *B. vulgatus* species was compared against *B. vulgatus* cut-off value that was previously established in our previous study (related pair: WSS score > cut-off; unrelated strain pair: WSS score < cut-off)^6,42^. Full details of the analysis procedure can be found in Koo et al.^20^.

### Analysis of growth dynamics of microbes

We have applied the Growth Rate InDex— MetaGenomic (GRiD-MG) approach to the three data sets to estimate the growth rates of microbes in a community^27^. The metagenomics reads mapped with *B. vulgatus* 1001283B150304-161114-D8 reference genome from the two data sets (Aggarwala et al., and Hourigan et al.) were mapped against the GRiD-MG database. The mapped reads were re-assigned using Pathoscope^43^ with default parameters. To be consistent with the WSS analysis, we showed GRiD scores for only *B. vulgatus* species for each analyzed sample.

### Comparative gene analysis

For comparative gene analysis, each donor sample from three data sets was used to align with *B. vulgatus* 1001283B150304-161114-D8 (NCBI accession number: GCF_015555055.1) using BWA^40^. Aligned reads from each reference genome were then sorted and indexed using SAMtools^44^. The resultant bam file was then converted to FASTQ format using BEDTools^45^. Each converted FASTQ file was then assembled using MEGAHIT and the resultant contig file was selected for annotation using Rapid Annotation using Subsystem Technology (RAST) server (https://rast.nmpdr.org/) with a default annotation scheme option, RASTtk^46^. The RAST server is based on the SEED data (http://www.theseed.org/). To construct a list of reference genes for comparative analysis, the same reference genome of *B. vulgatus* 1001283B150304-161114-D8 was mapped with the donor 283B metagenomics samples, assembled, and annotated using the above-mentioned procedures. Annotated donor 283B metagenomic sample was then used to compare with each annotated metagenomics donor’s samples using the “sequence-based” options implemented in the RAST server.

To determine significant differences between early and late donors from Aggarwala et al. and Hourigan et al. data sets, we have conducted statistical analysis on a total of 4911 genes. To do this, a total of the genes between the two groups were compared using ANOVA (Analysis of variance) with Benjamini–Hochberg FDR correction using STAMP (Statistical Analysis of Metagenomic Profiles)^47^.

### Ethics approval and consent to participate

Not applicable. We obtained the publicly available original sequence files from the publicly available sites.

### Supplementary Information

Supplementary Legends.

Supplementary Tables.

## Data Availability

The original sequencing data set of the samples used in this study was downloaded from the NCBI (accession numbers: PRJNA637878 for Aggarwala et al., PRJNA525458 for Hourigan et al.) and https://portal.hmpdacc.org/ for the HMP data set.
