# Supplementary material for: Identification of donor Bacteroides vulgatus genes encoding proteins that correlate with early colonization following fecal transplant of patients with recurrent Clostridium difficile
Source: Sci Rep. 2023 Aug 29;13:14112. doi: 10.1038/s41598-023-41128-y (PMC10465488; doi:10.1038/s41598-023-41128-y)
Supplement: Supplementary file 1 — Supplementary Legends. [file 41598_2023_41128_MOESM1_ESM.docx]

Supplemental Table Legends for

“**Identification of donor *Bacteroides vulgatus* genes encoding proteins that correlate with early colonization following fecal transplant of patients with recurrent *Clostridium difficile***”

Hyunmin Koo and Casey D. Morrow

**Supplemental Tables 1-6 in the Excel file (.xlsx)**

Supplemental Table 1: Raw sequence read information from Aggarwala et al. (A) single donor – single recipient, (B) single donor – multiple recipients, and from Hourigan et al. (C) donor-recipient pairs. The original sequence files were preprocessed, and deposited by both Aggarwala et al. and Hourigan et al.

Supplemental Table 2: Sequence depth and coverage from Aggarwala et al. and Hourigan et al. against reference *B. vulgatus* genome.

Supplemental Table 3: The results from the Growth Rate InDex – MetaGenomics (GRiD-MG) analysis on the Aggarwala et al. and Hourigan et al. The GRiD scores indicate the growth rate of *B. vulgatus* represented in the community.

Supplemental Table 4: WSS results on donors from Aggarwala et al. and Hourigan et al. All pairwise comparisons were conducted for all donors. The resultant WSS scores are represented as a numerical value. WSS scores that were above the cut-off (CO) value of 95.1, representing related B. vulgatus 1001283B150304 161114 D8 strain.

Supplemental Table 5: Statistical Analyses. The gene differences between early (283B, 1001271B, and D16) and late (1001275B, 1001175B, 1001217B, D06, and D15) donors were compared through ANOVA (Analysis of variance) with Benjamini-Hochberg FDR correction using STAMP (Statistical Analysis of Metagenomic Profiles).

Supplemental Table 6: From the comparative gene analysis, we found 4911 genes that found in all samples from Aggarwala et al. and Hourigan et al.
